# “Take services to the people”: strategies to optimize uptake of PrEP and harm reduction services among people who inject drugs in Uganda

**DOI:** 10.1186/s13722-024-00444-y

**Published:** 2024-02-23

**Authors:** Brenda Kamusiime, Kristin Beima-Sofie, Nok Chhun, Alisaati Nalumansi, Grace Kakoola Nalukwago, Vicent Kasiita, Chris Collins Twesige, Ritah Kansiime, Timothy R. Muwonge, Peter Kyambadde, Herbert Kadama, Peter Mudiope, Sara Glick, Barrot Lambdin, Andrew Mujugira, Renee Heffron

**Affiliations:** 1 Infectious Diseases Institute, Makerere University, Kampala, Uganda; 2 Department of Global Health, University of Washington, 3980 15th Ave NE, Box 351620, Seattle, WA 98195 USA; 3 Most-At-Risk Populations Initiative (MARPI), National STI Control Unit, Kampala, Uganda; 4 Ministry of Health, Kampala, Uganda; 5 Research Triangle Institute, Berkeley, USA; 6 University of California San Francisco, San Francisco, USA; 7 Department of Medicine, University of Alabama at Birmingham, Birmingham, USA; 8 Department of Medicine, University of Washington, Seattle, USA

**Keywords:** People who use drugs (PWUD), People who inject drugs (PWID), Harm reduction services, Pre-exposure prophylaxis (PrEP), HIV prevention

## Abstract

**Background:**

People who inject drugs (PWID) are at increased risk of HIV acquisition and often encounter barriers to accessing healthcare services. Uganda has high HIV prevalence among PWID and lacks integrated pre-exposure prophylaxis (PrEP) and harm reduction services. Understanding PWID experiences accessing and using harm reduction services and PrEP will inform strategies to optimize integration that align with PWID needs and priorities.

**Methods:**

Between May 2021 and March 2023, we conducted semi-structured interviews with PWID in Kampala, Uganda. We recruited participants with and without previous experience accessing harm reduction services and/or PrEP using purposive and snowball sampling. Interviews were audio recorded, translated, and transcribed. We used thematic analysis to characterize motivations for uptake of harm reduction and HIV prevention services, and strategies to optimize delivery of needle and syringe programs (NSP), medications for opioid use disorder (MOUD), and PrEP.

**Results:**

We conducted interviews with 41 PWID. Most participants were relatively aware of their personal HIV risk and accurately identified situations that increased risk, including sharing needles and engaging in transactional sex. Despite risk awareness, participants described engaging in known HIV risk behaviors to satisfy immediate drug use needs. All reported knowledge of harm reduction services, especially distribution of sterile needles and syringes, and many reported having experience with MOUD. Participants who had accessed MOUD followed two primary trajectories; limited resources and relationships with other PWID caused them to discontinue treatment while desire to regain something they believed was lost to their drug use motivated them to continue. Overall, PrEP knowledge among participants was limited and few reported ever taking PrEP. However, participants supported integrating PrEP into harm reduction service delivery and advocated for changes in how these services are accessed. Stigma experienced in healthcare facilities and challenges acquiring money for transportation presented barriers to accessing current facility-based harm reduction and HIV prevention services.

**Conclusions:**

Meeting the HIV prevention needs of PWID in Uganda will require lowering barriers to access, including integrated delivery of PrEP and harm reduction services and bringing services directly to communities. Additional training in providing patient-centered care for healthcare providers may improve uptake of facility-based services.

**Supplementary Information:**

The online version contains supplementary material available at 10.1186/s13722-024-00444-y.

## Introduction

The United Nations World Drug Report estimates that approximately 11.2 million people currently inject drugs, with 1.4 million of them living with HIV [1]. Globally, people who inject drugs (PWID) are 35 times as likely to acquire HIV as people who do not inject drugs [2]. In Uganda, HIV prevalence among PWID is 17% [3], which is significantly higher than the 5.1% prevalence in the general population [4, 5]. Moreover, PWID often belong to multiple key population groups, such as men who have sex with men, or engage in high-risk sexual activities to finance drug purchases, which further increases HIV risk [6]. In a pilot study of 67 women who engage in transactional sex who also inject drugs in Uganda, HIV prevalence was 31.3% [7]. High HIV prevalence among PWID in Uganda is largely attributed to lack of access to harm reduction services, including new injection equipment [8, 9].

Harm reduction interventions focusing on decreasing risks without advocating abstinence from drug use have proven effective in reducing HIV transmission among PWID [10, 11]. Examples of successful programs include needle and syringe programs (NSP) that prevent the sharing of injecting equipment and medications for opioid use disorder (MOUD) which aim to minimize drug use among those using opioids [12]. In Uganda, the Narcotic Drugs and Psychotropic Substances (Control) Act, 2016 regards drug possession and trafficking as unlawful activities [13]. Despite this, the laws that classify drug possession as a criminal offense are akin to those governing sex work, yet these regulations do not hinder the delivery of services for individuals involved in sex work. The Act provides for a “center to provide for the care, treatment and rehabilitation of persons addicted to narcotic drugs or psychotropic substances,” but it is underfunded and poorly utilized.

The Ugandan Ministry of Health (MOH) recently included NSP and MOUD harm reduction services in their HIV prevention guidelines as key strategies for reducing HIV transmission among PWID in Uganda [14]. Individuals receiving MOUD are administered a daily regimen of methadone syrup or buprenorphine pills as part of their maintenance therapy for a period of approximately 1–2 years, alongside individual and group psychotherapy sessions. HIV pre-exposure prophylaxis (PrEP), which effectively prevents HIV acquisition among those with high HIV risk, including PWID, was introduced into Ugandan HIV prevention guidelines in 2016. The U.S. President’s Emergency Plan for AIDS Relief (PEPFAR Uganda) provides free PrEP, MOUD, and NSP services through local implementing partners and the Ugandan MOH plays a key role in coordinating these partners and their programs.

PrEP eligibility in Uganda includes, but is not limited to, consideration of higher risk sexual behaviors (engaging in transactional sex, having multiple sexual partners of unknown HIV status), having multiple sexually transmitted infections in the past year, serodiscordant relationships where the partner is not virally suppressed, injection drug use, and frequent use of HIV post-exposure prophylaxis. National PrEP program data indicate that, as of December 2022, a total of 20,099 PWID had been tested for HIV, with 11,867 (59%) meeting PrEP eligibility requirements [15]. Of those eligible, 9,239 (78%) initiated PrEP, but only 1,554 (17%) refilled their PrEP prescriptions [15], indicating a large gap between policy intentions and uptake among this key population. Integrating PrEP into NSP and MOUD programs could facilitate increased uptake and adherence of PrEP among PWID [16, 17], optimize harm reduction service delivery, and further decrease HIV transmission in this key population [17, 18]. Better understanding of the motivations driving uptake of both harm reduction and HIV prevention services, including PrEP, may inform delivery strategies optimized for PWID. We conducted qualitative interviews with PWID to gain a deeper understanding of their experiences utilizing harm reduction and PrEP services as part of formative research for an implementation science study on PrEP delivery within harm reduction services for people who use drugs (PWUD) in Uganda.

## Methods

### Study design and population

We conducted a formative qualitative assessment to guide PrEP delivery within MOUD and NSP services for PWID in Kampala, Uganda.

### Setting

Our study includes 5 harm reduction sites in Kampala, Uganda: Butabika National Referral Mental Hospital, Most-At-Risk Population Initiative (MARPI), Uganda Harm Reduction Network (UHRN), Hope and Beyond rehabilitation center, and Serenity Center. In collaboration with funding from PEPFAR-Uganda, Butabika National Referral Mental Hospital opened a medication-assisted treatment (MAT) clinic in September of 2020, catering specifically to individuals with opioid use disorder (OUD). This is currently the only MAT clinic in the country. Eligibility for MAT requires meeting three criteria: (1) Being diagnosed with OUD according to DSM-5 standards; (2) Commitment to attending daily treatment at the MAT clinic; and (3) History of injecting drug use within the last 6–12 months. The medications currently prescribed at this clinic are Methadone and Buprenorphine. The other four harm reductions sites offered NSP services and psychotherapy, depending on the specific site.

### Data collection

Between May 2021 and March 2023, we used a combination of purposive and snowball sampling to recruit PWUD accessing harm reduction services from one of the five harm reduction sites or visiting Kisenyi and Luzira “hotspots” (i.e. locations frequented by PWUD where drugs were purchased and used). Individuals were eligible to participate if they were ≥ 18 years, HIV negative by self-report, currently or recently using recreational drugs, and willing to provide informed consent. Staff from harm reduction sites assisted with recruitment by identifying an initial subset of eligible PWUD from patient registers, who were introduced to the study team for consenting and interviews. After an initial round of qualitative interviews, an additional set of participants were purposively recruited to capture PWUD who had experience taking PrEP. Study staff received assistance from MARPI staff, a harm reduction site which provides services to PWUD using clinic- and community-based strategies, to facilitate hotspot entry and connect with hotspot leaders. Hotspot leaders helped identify and screen potential participants for eligibility and referred eligible participants to study staff. At the end of each interview, PWUD were asked if they knew additional peers who may be interested and eligible to participate. PWUD received compensation of 10,000 Uganda shillings ($2.60) for each participant they recruited who was successfully enrolled in the study. To focus exclusively on PWID, participants who did not describe injection drug use were excluded from this analysis.

Each participant took part in a single in-person qualitative interview guided by a semi-structured discussion guide (Additional file 1). The guide was developed collaboratively by the research team based on literature reviews, previous experiences working with PWUD and PWID populations in other settings, and knowledge of existing PrEP and harm reduction service delivery models and programs [19–24]. Participant interviews covered: (1) drug use experiences, (2) harm reduction knowledge and experiences, (3) HIV risk and risk perception, (4) knowledge and use of oral PrEP, and (5) perspectives of COVID-19 mitigation measures. Interviews were conducted in Luganda (local language) or English by three female and two male trained Ugandan social scientists. All interviewees, including those identified from “hotspots”, were interviewed at harm reduction sites for purposes of privacy and to ensure participant and interviewer safety. Interviews lasted a median of 46 min (range 28–72 min), were audio recorded with permission, and transcribed and translated (when necessary) into English by the interviewer, who also reviewed transcripts against audio for data quality. Participants received an IRB-approved reimbursement of 30,000 shillings ($8).

### Data analysis

We synthesized participant experiences to characterize drug, harm reduction service, and PrEP use motivations and barriers and provided recommendations for how to optimize PrEP and harm reduction service delivery for this key population in Uganda. Using a thematic analysis approach [25, 26], an initial codebook was developed by five members of the research team (AN, BK, KBS, NC, and CCT) through open coding a subset (n = 10) of full transcripts to identify key concepts related to drug use, harm reduction, HIV risk and HIV prevention. The codebook was further refined by reviewing additional transcripts (n = 6), testing the codebook, and revising codes and code definitions via group discussions. Four coders (AN, BK, NC and KBS) used the final version of the codebook to independently code each transcript. Each coded transcript was then reviewed by another member of the coding team to evaluate code agreement and identify discrepancies. All coding discrepancies were resolved through team discussion to achieve consensus. Queries and code co-occurrence reviews were used to extract participant descriptions of how drug use experiences impacted decisions to engage in harm reduction and HIV prevention behaviors and services, as well as identify recommendations for optimizing PrEP and harm reduction service delivery. *ATLAS.ti* software (version 23) was used to facilitate data management and analysis.

## Results

Of the 50 PWUD who participated in interviews, nine were ineligible for this analysis because they did not report injecting drugs. Most were male (63%), with a median age of 29 years (interquartile range [IQR]: 25, 33). They reported a median of 8 years of drug use experience (IQR: 5, 13). The most frequently used drugs were heroin (76%), followed by cocaine (37%) and marijuana (37%) (Table 1).

**Table 1:** Demographic characteristics of in-depth interview participants who inject drugs, Kampala, Uganda, 2021–2023 (N = 41)

Characteristic	N (%) or Median [IQR]
Program recruitment sites	
Clinic-based	32 (78)
Hot-spot	9 (22)
Gender	
Men	26 (63)
Women	15 (37)
Age (years)	29 [25, 33]
Relationship status	
Single	15 (37)
Partnered/Married	18 (44)
Divorced/Separated/Widowed/Widower	8 (19)
Highest level of education	
University/college	14 (34)
Secondary	18 (44)
Primary	9 (22)
Occupation	
Has income-generating activity^1^	28 (68)
Unemployed	10 (25)
Student	3 (7)
Ever tested for HIV	41 (100)
No. of years of drug use	8 [5, 13]
Most used drugs^2^	
Heroin	31 (76)
Cocaine	15 (37)
Marijuana	15 (37)
Other	23 (56)
Use of needle and syringe program (NSP)	
Yes, regularly (almost every time come to the clinic)	13 (32)
Yes, rarely (about once every three weeks/once a month)	7 (17)
No	21 (51)
In the last year^3^	
Used a syringe that had already been used by someone else	
Every day	4 (20)
A few times a week	2 (10)
A few times a month	0 (0)
A few times a year	7 (35)
Never	7 (35)
Let someone else use a syringe that you had already used	
Every day	3 (15)
A few times a week	6 (30)
A few times a month	0 (0)
A few times a year	4 (20)
Never	7 (35)

^1^Income generating activities as defined by participants include boda boda driver, hairdresser, businessperson, trader, sex worker, fisherman, shoemaker, physician, musician, etc.

^2^Open-ended question about what drugs commonly used; PWID gave multiple responses, as a result percentage total does not equal 100; Other category includes crack, crystal meth, pethidine, cigarettes, etc.

^3^Among those that responded yes (N = 20)

Almost all participants could name high HIV risk activities and had accurate perceptions of their own HIV risk. About half described engaging in transactional sex, often without condoms, to get money to purchase drugs. Others described sharing injection equipment as their primary source of HIV risk, sharing mainly when new equipment was not readily available and they were experiencing withdrawal symptoms.

*“You know in the ghettos, it is hard not to share syringes because even when the health workers give you many syringes, they get finished and you are left with no option but to share.”* – [Male, age 30]

PWID were also well-aware of available harm reduction services describing both inpatient rehabilitation programs and drop-in service centers with access to clean injection equipment and MOUD. Participants reported learning about harm reduction services from healthcare workers (HCWs) who visited their communities, peers who had accessed services themselves, or friends and family invested in helping them address their addiction. All participants recognized the importance of harm reduction and HIV prevention services for their community.

*“[W]e do need these services. Like I told you, we are at risk of HIV. We need these items to use to prevent ourselves from HIV.”* - [Female, age 38]

Despite high awareness, participants identified barriers preventing them from consistent access to HIV prevention and harm reduction services, and recommendations for how to optimize PrEP and harm reduction service delivery (Table 2).

**Table 2:** Barriers preventing consistent access to PrEP and harm reduction services and recommendations to optimize delivery among PWID in Kampala, Uganda

Theme	Description	Illustrative quotes
Drug use influenced PWID behaviors	Stealing money was a strategy used to support drug access	*“Because of the constant need to use heroin, you end up stealing to get money to buy the drug because each time you use heroin, you need more so that you do not get turkeys.” –* [Male, age 37]
	Drug use facilitated increased drug use to address developed tolerance	*“The more you use the drug, you find that your brain gets used to it, and the drug no longer makes you high, and yet you need to get more “heights” (more effects of the drug) than what you are getting. That is what in most cases causes us to change from the drug that you were taking to use another drug.” –* [Male, age 27]
	Withdrawal symptoms made it challenging to keep consistent employment	*“There is a woman who gave me a job but stole from her because when the turkeys come in, you cannot think straight. You would rather steal than have turkeys. They are so painful and unbearable.” –* [Female, age 20]
	HIV prevention was not prioritized when PWID were experiencing withdrawal symptoms	*“[W]e would divide ourselves into groups and then share [injection equipment]. I know I was at high risk of contracting HIV because most of my friends were prostitutes and yet some were HIV positive. But during that time when you need to treat the turkeys, you cannot think of that.”* – [Female, age 23]
Poor utilization of HIV testing services despite high HIV risk awareness	HIV negative test results encouraged PWID to engage in less risky behaviors	*“One day I went to [the hospital] and tested for HIV, and my results were negative. Remember, I was injecting myself and sharing the syringes with my friends so when my HIV results came back negative, the thought of only using my foil crossed my mind, and realized that it was even safer.”* – [Male, age 31]
	Beliefs about already being HIV positive delayed or prevented HIV testing	“*When the health worker drew blood from me [for an HIV test], while waiting for the results, I was in fear and was positive that my results would be positive….There are junkies among us whom we knew were HIV positive but we still shared syringes with them. They would use syringes and then you also use them. I was happy when the health worker told me that I am HIV negative.”* – [Male, age 30]
Low PrEP literacy hindered PrEP uptake and adherence	Confusion about how PrEP worked influenced strategies for use	*“I think [taking PrEP everyday] is not possible because you will not have sex with the HIV infected person every day. What is better is to take that medicine when you expect that you are going to have sex with such a person. That is because they told us that medicine remains in the body for five days; that if you swallow a PrEP tablet today, it remains in the body for five days. So, for all those days if you have sex with someone who is infected with HIV, you do not contract it.”* – [Male, age 47]
	Limited access to food was described as a barrier to consistent PrEP use	*“I would only take it once in a while and yet you have to have eaten something before taking PrEP, and even after taking PrEP, you had to eat a lot. I did not have money to buy what to eat and it is hard to eat when you are using drugs…..That became a barrier for me to adhere to PrEP.”* – [Female, age 23]
	PrEP was used in relation to perceived risk	*“I do not take PrEP every day because I do not have sex every day. I only take PrEP when I am going to have sex. Why then should I be taking PrEP if I am not having sex?”* – [Male, age 28]
MOUD uptake was motivated by a desire to restore what was lost	Reconnecting with family was a motivator for initiating methadone treatment	*“I wished for the grounds to swallow me and disappear for good. I reflected on my life and indeed I was not the best mother to my children. I had let them down. That was my turning point to stop using drugs.”* – [Female, age 46]
	Methadone helped PWID feel positive about their appearance	*“Coming for methadone has been so helpful to me because I am now back to who I used to be. I barely recognized myself before because I never used to shower, I never cut my hair at all, and I was always dirty and disgusting. When I started taking methadone, I started changing gradually. I started bathing and even went for a haircut. I started putting on clean clothes. When I look at myself, I feel I am back to who I am.”* – [Male, age 37]
	Being sober positively influenced life goals	*“Now that I am sober, I do not want to even have friends who stay in the ghetto. I do not admire that life now. I know what I want to be in life…..I want to live a normal life, I want to go back to school, I want to be like other girls.”* – [Female, age 23]
PWID preferred decentralized services and peer delivery	Transportation to clinics and complicated enrollment processes are challenges to uptake	*“[H]ealth workers came and told us about MAT…They said ‘we do not deny that people use drugs, it is true, however if there is anyone who wants to stop using drugs, come.’ We were very happy when we heard them…..They left us with a phone number and said that this is their doctor’s number, “anyone who wants… the number is there”. The first person who called the number was told “I am in Butabika, that is where you should find me”. However, you could not just go directly to Butabika, you had to first come to this place and they make for you a file. Then you take that file to Butabika.”* – [Male, age 42]
	Long wait times and client-provider interactions posed challenges to facility-based service delivery for PWID	*“The issue of going to the hospital gives us some difficulty but if they put the medicine at harm reduction [sites] it’s very easy……because these [providers] of harm reduction, they understand us and when they come, they treat us as people rather than at the hospitals. At times you reach there with an appointment but you still have to make a line and you may get thirsty for the drug while you are there. But if it is here [at the harm reduction site] they know my appointment and work on me immediately…”* – [Female, age 26]
	Co-location of services facilitates adherence through improved convenience	*Getting my PrEP from where I get MAT is good because it is easier to access. And in case you want to know anything, it is easy to access the health workers and ask them anything that you want. I do not have to incur transport costs because going to [another location] to access PrEP is really expensive. It would be hard for me to get the refills from [another location], and so I am happy that I was given the chance to pick-up PrEP from where I am getting methadone from.”* – [Male, age 31]
	Peer delivery decreases stigma and improves accessibility	*“Of course, the peers have the most access to us to provide PrEP to us because they can come to the community and find us there.”* – [Male, age 30]

## Drug use influenced PWID behaviors

Most participants described that initial drug use was driven by peer pressure or curiosity, while continued use was motivated by needing to cope with life stressors, filling a perceived void, appreciating positive feelings experienced while using, and experiencing severe withdrawal symptoms (“turkeys”) when not using. All participants reported reaching a stage of daily use and a shifting mentality towards reliance on drugs to function.

The primary limitation on drug use frequency was availability of funds. Although the majority (68%) of participants reported having some form of income generating activity, most did not report consistent employment and described negative changes in behavior in order to access finances needed to purchase drugs. Participants described how once they started using drugs, their priorities shifted to focus solely on how they could access their next fix.

*“I would steal to get money. I also sold sex. I had to do all that to get money to deal with my turkeys. It is a terrible feeling when you have turkeys…when I inject myself with heroin, I feel better but as I am injecting myself I am already thinking of where I am going to get the money for the next fix.”* – [Female, age 20]

## Poor utilization of HIV testing services despite high HIV risk awareness

Despite recognition of being at increased risk for acquiring HIV, participants noted rarely testing for HIV. They reported testing when services were taken to them or they were in a health facility for another reason and offered a test. Many shared experiences of engaging in high HIV risk activities, or becoming sick, which caused them to assume they had HIV. This led to testing delays out of fear of confirming their presumed positive status. Most were surprised upon working up courage to test and receiving HIV negative results.

*“When I was tested and told that I am HIV-negative, I was surprised and could not believe it because I used to have unprotected sex. I promised myself that I would never risk my life like that ever again.”* – [Male, age 28]

## Low PrEP literacy hindered PrEP uptake and adherence

PrEP knowledge was limited among participants and most were not currently taking PrEP. Those taking PrEP had learned about PrEP from HCWs, rather than through peers or community messaging, and had been motivated to start PrEP in order to remain HIV negative despite continued engagement in high HIV risk behaviors.

*“I told my boyfriend that I need to take PrEP to protect myself from contracting HIV since I sometimes go and hustle because we need money to survive…and he does not have a job.”* – [Female, age 20]

Participants using PrEP reported varied use patterns and several misconceptions about how PrEP worked. Some participants believed PrEP treated other illnesses and illness symptoms, including other STIs, fevers, and stomach aches, and not just HIV.

*“[F]or me, if I get some kind of fever, I take PrEP. That is why, I do not want to run out of PrEP tablets. This is because I discovered that it also treats and cures other illnesses.”* – [Male, age 47]

Participants mentioned many commonly known barriers to PrEP adherence, including large pill size, challenges remembering to take a daily pill, and fear of stigma given confusion by others about the differences between antiretroviral treatment (ART) for HIV and PrEP. A few participants were also concerned about potential PrEP and MOUD drug-drug interactions. Several participants taking PrEP and struggling with adherence mentioned access to food as a barrier.

*“There are days when I miss taking PrEP because of a lack of what to eat and I fear taking the pill on an empty stomach.”* – [Male, age 37]

When discussing their PrEP use, some participants disclosed that they shared PrEP with others, including friends believed to be at high risk or those lacking transportation to health facilities for their own prescription. Two participants described getting PrEP from multiple facilities with the intent of selling it for profit. Others took PrEP on demand, just prior to sexual activity.

*“I will not say that I take PrEP every day like the health workers told me to. There are days when I miss taking the pill but take it whenever I am going to have sex with my girlfriend.”* – [Male, age 28]

Others avoided taking PrEP when planning to engage in transactional sex, fearing that PrEP would negatively impact sexual performance, viewing this as problematic since they relied on sex to earn a living.

*“I use condoms sometimes. I tried PrEP but I stopped it because it was making me uncomfortable. PrEP makes me weak, it makes me feel sleepy and also the urge for sex completely goes down yet I have to have sex in order to survive. Once you do not have the urge, your private parts will not be lubricated and it will be hurting. Secondly, its even going to be more risky for the condom to break……I took PrEP for just a week but I realized I can’t manage it.”* – [Female, age 38]

## MOUD uptake was motivated by desire to restore what was lost

PWID currently engaged in MOUD described reaching a point where they were ready to stop using drugs. They recognized a loss of something they valued, whether that was relationships, independence, or health and wellness, and were motivated to get it back. For some participants, this realization came from witnessing positive changes in friends or peers who had joined MOUD.

*“In the beginning, I loved my drugs but later realized that I had lost so many things at home…..I heard about MAT and saw that my friends who had joined MAT had started to change. I asked them how I can join MAT… Since I was serious about stopping to use drugs, I accepted to be on medication and forgot about using drugs.”* – [Female, age 20]

For others, they reached a point where they wanted to repair a broken relationship or build a new relationship, especially participants who were parents and not currently taking care of their children. Other participants were motivated to engage in treatment because they recognized the strain drug use placed on their physical or emotional health. Participants had seen others experience negative health outcomes, such as acquiring HIV or dying from an overdose. Others were tired of the physical and emotional toll experienced during withdrawal. Some participants described being so motivated to access MOUD that they started injecting, rather than smoking, drugs in order to qualify for services.

*“Ever since the MAT program started, they did not want (to enroll) people who use drugs taken with the “foil” [consumption by inhalation] method. They were only giving that drug (methadone) to someone who was using injectable drugs. I told myself, as someone who was determined and I really wanted it, I decided to start using the injection so that I can be able to join the MAT program….so that I could restore my life back to normal.”* – [Male, age 47]

## PWID preferred decentralized services and peer delivery

The most cited barrier to accessing harm reduction services and PrEP was lack of funds for transportation to facilities. Accessing MOUD was especially challenging given it was only available at one site and required a two-step enrollment process that required transportation between two different clinics. Participants felt that the best strategy to address transportation barriers was to deliver services in PWID communities.

*“The most important thing is bringing services closer to us. If services are put far away from where a drug user finds comfort, then they will not benefit from the services.”* – [Male, age 30]

Similarly, participants believed that integrating PrEP into harm reduction services would improve uptake of both services by providing a single access point. Participants already taking PrEP offered from harm reduction clinics appreciated how integrated delivery improved convenience and adherence.

*“I feel good because it is not difficult for me, as I do not have to go to another place to get my PrEP……when I come here, I get my MAT from one station and go to another or even at the same point I can get my PrEP.”* - [Male, age 30]

Negative interactions with HCWs and staff, especially security staff also discouraged participants from traveling to clinics for services. While many participants described positive interactions with harm reduction clinic staff, their journey through the health system prior to reaching those HCWs included encounters with staff at other clinics who were judgmental, rude, and condescending. PWID also associated HCWs with police. These negative interactions limited willingness to seek out facility-based services. One suggestion offered by participants was to train and use peers to help deliver services.

“*If the services are directly coming from [healthcare workers], drug users will say, ‘These are police informers, they are going to bring police to us’…..If it is someone they know who is using or was using drugs they can trust that [peer] and it is easier for them to get information from such a person*.” – [Female, age 38]

## Discussion

This qualitative study identified strategies that could facilitate uptake of PrEP and harm reduction services among PWID in Uganda. Participants shared important information related to HIV risk awareness, behavioral decision-making, and health services utilization that perpetuate high rates of HIV acquisition among PWID. Participants were aware they were engaging in high HIV risk activities, knew of strategies to reduce risks, and were willing to adopt risk mitigation approaches. However, experiencing withdrawal symptoms shifted priorities away from adopting risk prevention behaviors in circumstances where HIV prevention or harm reduction tools were not readily available. Participants suggested that increased availability and integration of harm reduction and HIV prevention services in their communities would improve service uptake.

Health behavior decisions affecting uptake of prevention services are often complex, influenced by a combination of facilitators, barriers and the interactions between them. Anderson’s Model of Health Services Utilization [27, 28] highlights how health services utilization is driven by the interaction between population characteristics and environment, both of which influence behavior. Aligned with this model, our results highlighted how a combination of pre-disposing characteristics (ex: health beliefs, knowledge of HIV transmission), enabling resources (ex: funds to travel to clinic, access to new injection supplies), and need (ex: value placed on remaining HIV negative, withdrawal symptoms) influenced immediate and longer-term behaviors affecting HIV acquisition. This aligns with previous research evaluating influences on PrEP use among PWID in the US [29], and highlights the need for PrEP implementation strategies that simultaneously address complex, interrelated barriers to ensure consistent adoption of harm reduction and HIV prevention behaviors.

Our study findings highlight the need for expanded community and clinic-based messaging on PrEP. The Information, Motivation and Behavior model [30, 31] describes how information acts as a precursor for developing motivation to adopt prevention behaviors, including PrEP. Participants in our study had limited and sometimes inaccurate knowledge of PrEP, potentially decreasing motivation for PrEP use. In addition, participants described varied patterns of use outside current recommendations, including sharing PrEP pills with others and only taking PrEP pills immediately prior to engaging in sexual activity. While this strategy of event-driven PrEP is recommended for MSM [32, 33], this recommendation has not yet been extended to other populations. These alternative use patterns illustrate how partially correct information could motivate use, but may lead to ineffective prevention.

Other studies have also identified limited PrEP awareness or inaccurate information on PrEP among PWID [34–38]. To improve awareness, one study leveraged existing rapport with harm reduction providers to improve accuracy of PrEP knowledge among PWID [35]. Participants in our study reported having strong relationships with harm reduction staff, making a similar approach of providing education through harm reduction staff a potential strategy for improving PrEP knowledge. Participants also mentioned being highly influenced by peers, joining MOUD programs only after observing the positive impact of MOUD on peers’ lives or taking PrEP based on information shared by peers. As observed in other studies [39, 40], community education through peer leaders may be another strategy to increase PrEP knowledge and appropriate use.

PWID need harm reduction service delivery models that are flexible and patient-centered [41–44]. Transportation to health facilities prevented consistent uptake of both harm reduction and HIV prevention services among study participants. In addition, participants reported negative interactions with HCWs and clinic staff from non-harm reduction sites that further prevented PWID from seeking services at facilities. Low-barrier care (LBC) is a multi-component evidence-based intervention targeting populations that have been hard-to-engage in traditional HIV care programs [45]. Clinics implementing LBC have been successful at engaging PWID living with HIV by adapting services to directly address patient barriers to care, including providing transportation support and patient-centered counseling [45, 46]. Using a LBC approach for delivering harm reduction and HIV prevention services could improve uptake of facility-based services.

Concerningly, our study identified that restricting MOUD eligibility to those who inject drugs could influence some people who use opioids to transition into injection, a route of administration that carries higher levels of infectious disease and overdose risks [47]. Methadone treatment is a gold standard medication for treating OUD, and an OUD diagnosis is not dependent on the route of drug administration [48]. Given additional harms associated with drug injection, MOUD programs should ensure eligibility requirements are inclusive of all people with OUD, regardless of their route of administration. Doing so will optimize the potential health benefits by not only expanding the number of people who can access MOUD but also preventing people from adopting riskier drug use behaviors to become eligible under current guidelines.

This study has several limitations. Our findings are based on a sample of PWID located near Kampala, a large urban city in Uganda, and may not be generalizable to populations in other geographic areas. Our participants reported early initiation of drug use, most during adolescence, and our findings may not reflect the experiences of older aged PWID. We did not collect demographic information on sexual behaviors, PrEP use, or MOUD use, limiting our ability to describe these characteristics beyond what was reported by participants during interviews. In addition, our study was limited by lack of access to alternative substance use disorder (SUD) treatment services in Uganda and poor uptake of in-patient rehabilitation due to low willingness to be admitted, coupled with a shortage of rehabilitation centers within the country. We intentionally recruited participants from hotspots and used snowball sampling in order to include diverse perspectives on harm reduction services. However, to ensure well-being and confidentiality, all participants were required to travel to harm reduction sites for interviews, which may have led some PWID recruited from hotspots to decline participation.

## Conclusion

Ending the HIV epidemic will require improving uptake of HIV prevention services among key populations, such as PWID, with high rates of HIV acquisition. Our qualitative study suggests that while PWID are aware of harm reduction services, and have accurate perceptions of personal HIV risk, logistical and behavioral barriers prevent many from utilizing these services. In addition, many PWID in our study had limited PrEP knowledge. Optimizing uptake of harm reduction and HIV prevention services among PWID in Uganda will require adapting program delivery strategies to overcome existing barriers to better meet patient needs. Bringing services to communities and improving healthcare provider training may be strategies that improve adoption of HIV prevention behaviors, including PrEP. Future research should evaluate implementation of suggested strategies to determine their impact on service utilization and HIV acquisition among PWID.

### Supplementary Information

**Additional file 1.** In-depth interview guide.

## Data Availability

Data reported in this paper are available upon request.

## Abbreviations

PrEP: Pre-exposure prophylaxis
MOUD: Medications for opioid use disorder
NSP: Needle and syringe programs
PWUD: People who use drugs
PWID: People who inject drugs
