# Supplementary material for: “Take services to the people”: strategies to optimize uptake of PrEP and harm reduction services among people who inject drugs in Uganda
Source: Addict Sci Clin Pract. 2024 Feb 23;19:13. doi: 10.1186/s13722-024-00444-y (PMC10893723; doi:10.1186/s13722-024-00444-y)
Supplement: Supplementary file 1 — Additional file 1. In-depth interview guide. [file 13722_2024_444_MOESM1_ESM.docx]

**Supplementary Material: Semi-structured interview guide to elicit perspectives on accessing harm reduction services and HIV prevention services among PWUD in Kampala, Uganda**

**Guide Details:**

Version 1.0

August 26, 2020

**Interviewer Instructions:**

The following is a guide. Try to ask all the questions below in the order given, but it is more important to maintain the flow of discussion. Suggested probes have been included.

*************************************************************************************************************

**Before turning on the recorder, start with the following introductory script:**

*Hi, my name is ____________. Thank you for agreeing to participate in an interview today. You are participating in the PWUD study. In our conversation today we are going to talk about your experiences using drugs, harm reduction and HIV prevention. We hope to use the information you share with us to improve how we design programs that reduce harm and prevent HIV among people who use.*

*During our conversation I will ask you questions and you are free to answer in any way you wish. If anything is unclear, please feel free to ask me to explain it. I would like to record our conversation so I don’t miss anything that you say. I will not include your names on any documents or in the recording. Your responses will be kept confidential, which means we will keep what you say private from others. Is it okay if I record our conservation? [Wait for the participant to give verbal consent to recording]*

*Before we start I would like to remind you that there are no wrong answers during our discussion. We are interested in knowing what you think, so please feel free to be open and share your point of view.*

*If you want to stop the discussion at any time, just let me know. Do you have any questions for me before we get started? [Wait for participant to respond – answer any questions they have]*

*I am turning on the recorder now.*

*************************************************************************************************************

**Before beginning interview questions, please read the following script for the recording:**

*Today is [day of week], [month, day, year] and it is now [time of day]. This is interview [ID number] conducted at [discussion location].*

**You are now ready to begin asking the questions outlined below.**

**Background**

First, let’s get started by you telling me a little about yourself.

- Whom do you live with (children, partner)
  - What is it like living in your home?
  - How do you feel about your living situation?
- What activities do you do to generate income? Tell me about these activities

**Drug Use Experience**

Now, I’d like to hear about your drug use experiences. Can you tell me about what drugs you are using or have used in the past year? (probe to find out which drugs they are using)

- - When and how often do you use these drugs?
  - Where do you get your drugs? Where do you use your drugs (is it a private or public place)

(***For those who use drugs that requires a syringe***) Can you tell me about your syringe usage?

- How often do you re-use your syringe(s)?
- With whom do you share your syringe(s)?
- Where do you get syringes when you need more?

**Harm Reduction** (***skip this section for those who are not going to either clinic***)

Now, I’d like to learn more about your experiences with harm reduction services. Can you tell me how you ended up going to MARPI or Butabika Hospital?

- What was the experience like when you went to MARPI or Butabika Hospital?
- What kinds of information did the people working there talk with you about?
- How did you feel about your experience at MARPI or Butabika?
  - What did you like about the experience?
  - What didn’t you like about the experience?
- What services were you offered at this clinic? Probe about NSP and MAT if not mentioned by the participant.
  - If participant was offered NSP or MAT - How did you feel when you offered needles and syringes or MAT?
  - Did you end up accepting what was offered? Why or why not?
  - For those that accepted NSP: How often do you exchange the needles and syringes? How easy or difficult is it for you to access this service?
  - For those that accepted MAT: How often do you receive MAT? How easy or difficult is it for you to access MAT?
- What are your thoughts on the needle and syringe program? What are your thoughts on the MAT program?

**Risk and Risk perception**

We are now going to talk about HIV and the risk of getting HIV. First, I’d like to hear about your experiences with HIV testing. What happened the last time you tested for HIV?

- How often do you test for HIV and why? Probe to learn about previous HIV tests***(If the person has never tested ask them why they have never tested (barriers to HIV testing****)*
- What sorts of things might make it difficult for you to get tested for HIV regularly?

Now, I’d like to learn more about how you think about your risk for HIV. What do you think is your risk of contracting HIV currently? Why do you say so?

- If not answered by the previous question: What do you do currently that might put you at risk of contracting HIV? Probe about any previous mention of sharing needles, sex behaviors including unprotected sex, transactional sex and frequent sex with partners of unknown HIV status
- Have you always felt like this (I.e. at risk or not at risk)? If yes, why? If no, what has changed?
- When in your life did you feel most at risk of contracting HIV? Why?
- What do you do to protect yourself from contracting HIV?

**Oral PrEP**

- Now I would like to talk about HIV prevention. What have you heard about PrEP? (***if they do not know about PrEP, read explanation***): PrEP is the medication that someone who is HIV negative can take to reduce their risk of contracting HIV. If someone takes the medication every day, it has been shown to be very effective at protecting them from HIV even if they are exposed to the virus.

***(Skip bullets 1-4 for those that have no prior knowledge about PrEP***)

- Where did you learn about PrEP?
- Who do you think should take PrEP? Why do you say so? Probe to understand the populations they think are at risk (if he/she does not mention PWUD among those at risk, ask the question below)
- Do you know anyone who has taken PrEP?
- Have you ever taken PrEP or been offered PrEP? (**if yes skip to PrEP experiences**)
- Based on what you know/ what you have just learnt about PrEP, what do you think about the idea of taking a pill everyday to prevent HIV? Would that be something you would want to do?
- How interested would you be in taking PrEP? Why do you feel that way?
- Some people say taking the medication daily could be a challenge. How easy or difficult would it be for you to take medication on a daily basis?
- What do you think could help you to take medication on a daily basis?
- If you were to access PrEP, where would you want to access PrEP services from? Why do you say so?
- Who would you want to provide PrEP to you?

**PrEP experiences (Ask only for those who have been offered or taken PrEP)**

- Where were you first offered PrEP?
- What happened when you were offered PrEP?
- How did you feel about being offered the opportunity to take PrEP?
- What were you counseled about PrEP?
- Did you end up starting PrEP? Tell me the story of what happened.
  - What were your reasons for wanting to take PrEP?
  - What fears or concerns did you have about taking PrEP?
  - What was your experience like taking PrEP? (probe: were you able to continue PrEP, why or why not?; how could you be better supported to continue, ie: delivery locations, long-acting options, etc)

What do you think about PrEP being made available at the place where you receive your harm reduction services?

- - How would you want this service to be delivered?
  - Who would be the best PrEP provider?
  - Would you want to take PrEP home with you or leave it at the clinic and come to take a pill everyday as you are also getting your methadone?

**Perspectives on COVID-19**

We have been hearing a lot about the corona virus or COVID-19 lately, and I would like to get your perspective on the current situation. Would that be ok with you? *(wait for participant response).*

- Based on what you know or have heard, what is corona virus/COVID-19?
- What are people in your community saying about it?
  - How are you influenced by what they say?
  - Tell me about any stigma associated with the virus.
- In general, how has the situation with corona virus affected your daily life?
  - What is it like for you to move around (transportation)?
  - How has it influenced your ability to go to work or earn income?
  - What steps are you taking to keep yourself healthy and safe? How helpful are these measures, in your view?
  - Do you know anyone who has gotten COVID-19 illness or have you gotten it?What was their/your experience?
- Have the services being offered at the clinic changed or been modified since COVID-19? How so?
  - In what ways has COVID-19 affected your ability to access harm reduction services?
  - In what ways has COVID-19 affected your ability to access HIV prevention services?
  - How did you feel about being at the clinic with other clients during this time of social distancing (if applicable)?

As we finish talking today, is there anything about your experiences using drugs that you didn’t tell me about but think is important for me to know?

Is there anything about accessing or using harm reduction services (MAT or NSP) or HIV prevention (oral PrEP) that you think is important to tell me, but I didn’t ask about?

That is all of the questions I have for you today. Thank you for your time. If you do not have any further questions or comments, I will now turn off the recorder*.*
